# Supplementary material for: Dietary Supplementation of Microbial Dextran and Inulin Exerts Hypocholesterolemic Effects and Modulates Gut Microbiota in BALB/c Mice Models
Source: Int J Mol Sci. 2023 Mar 10;24(6):5314. doi: 10.3390/ijms24065314 (PMC10049499; doi:10.3390/ijms24065314)
Supplement: Supplementary file 1 [file ijms-24-05314-s001.zip › ijms-2192626-supplementary.pdf]

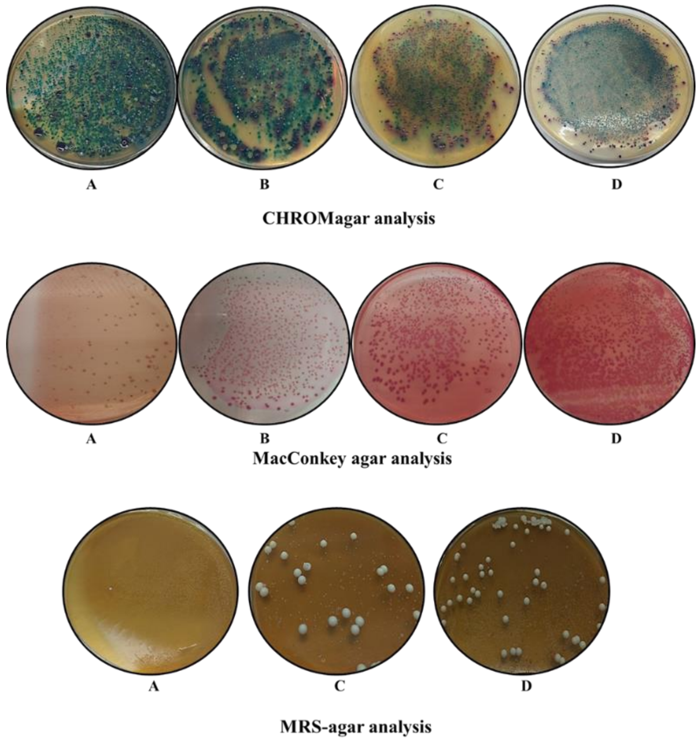

Figure S1. Analysis of intestianl microbiota of mice on various selective agar media inlcluding CHROMagar, Mackoncky agar and MRS agar. The legends are described as: A: control B: scratch control C: Dextran fed mice D: inulin fed mice.

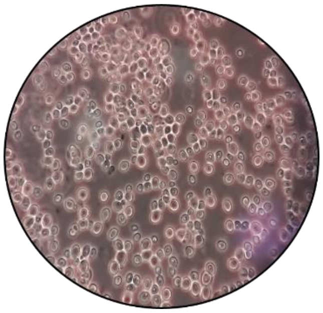

Figure S2. Microscopic image of large white colonies of yeast isolated from mice intestinal fluid appeared on MRS-agar plates.
